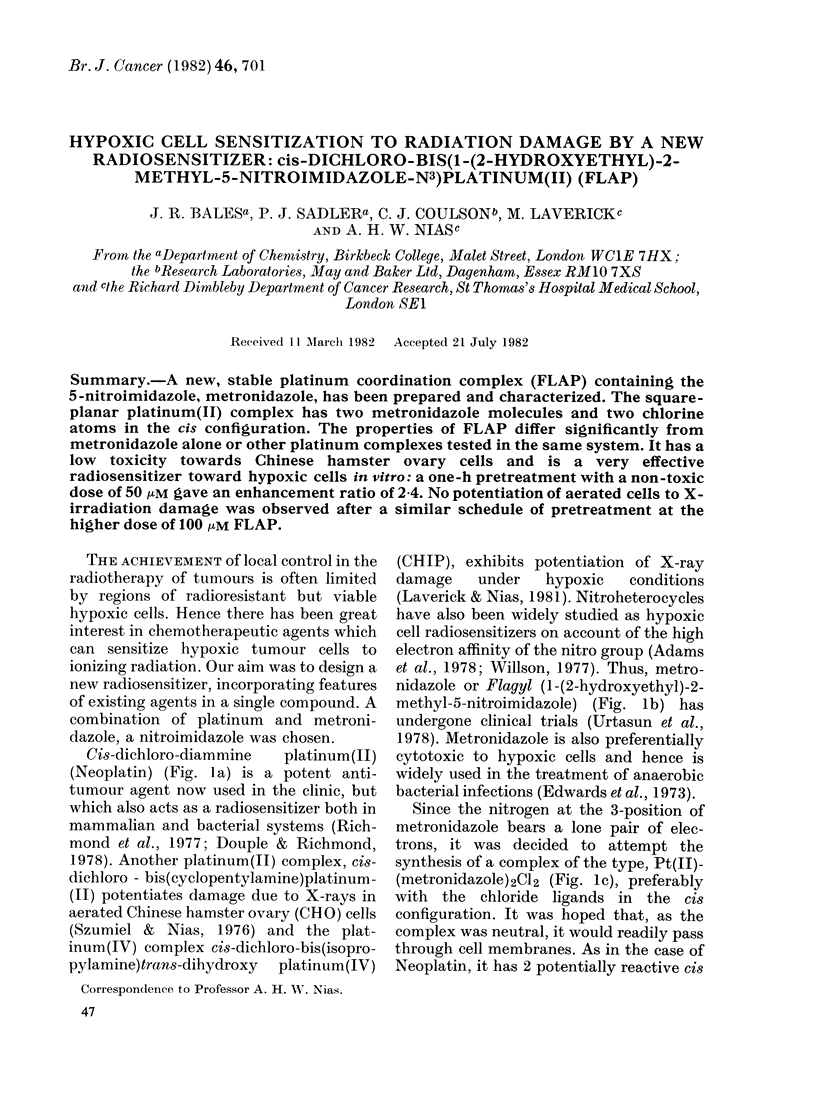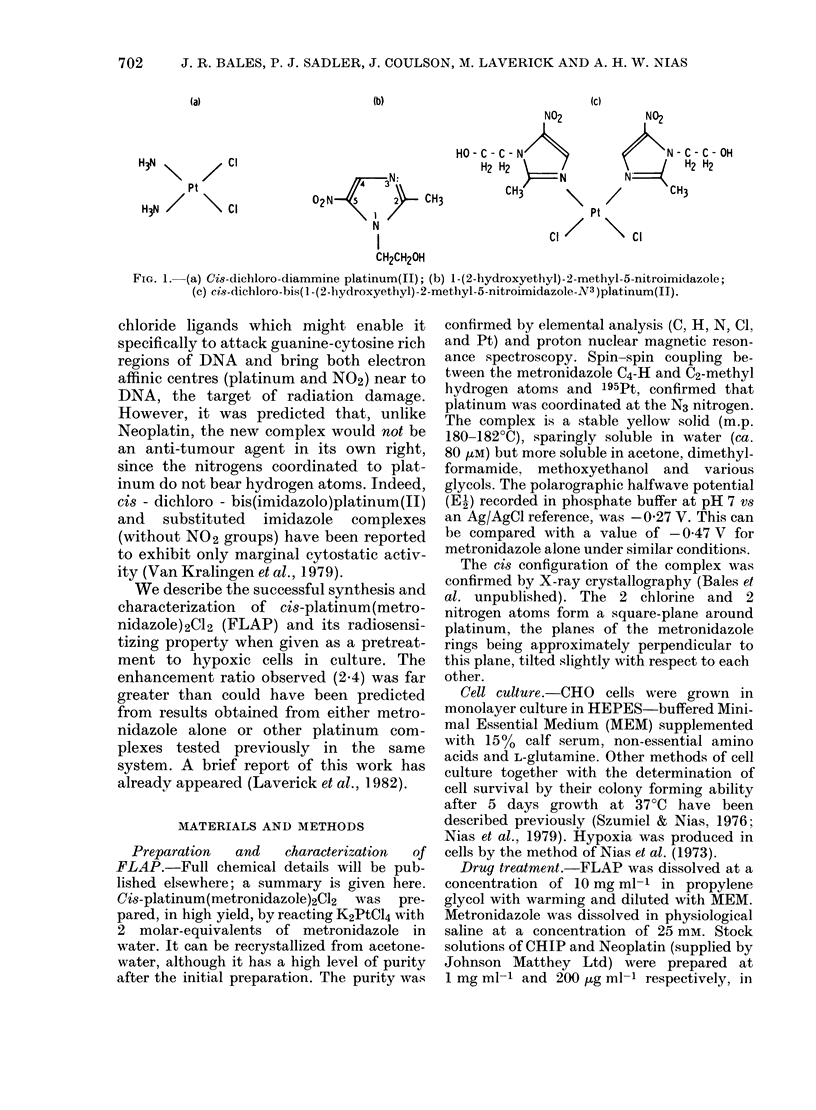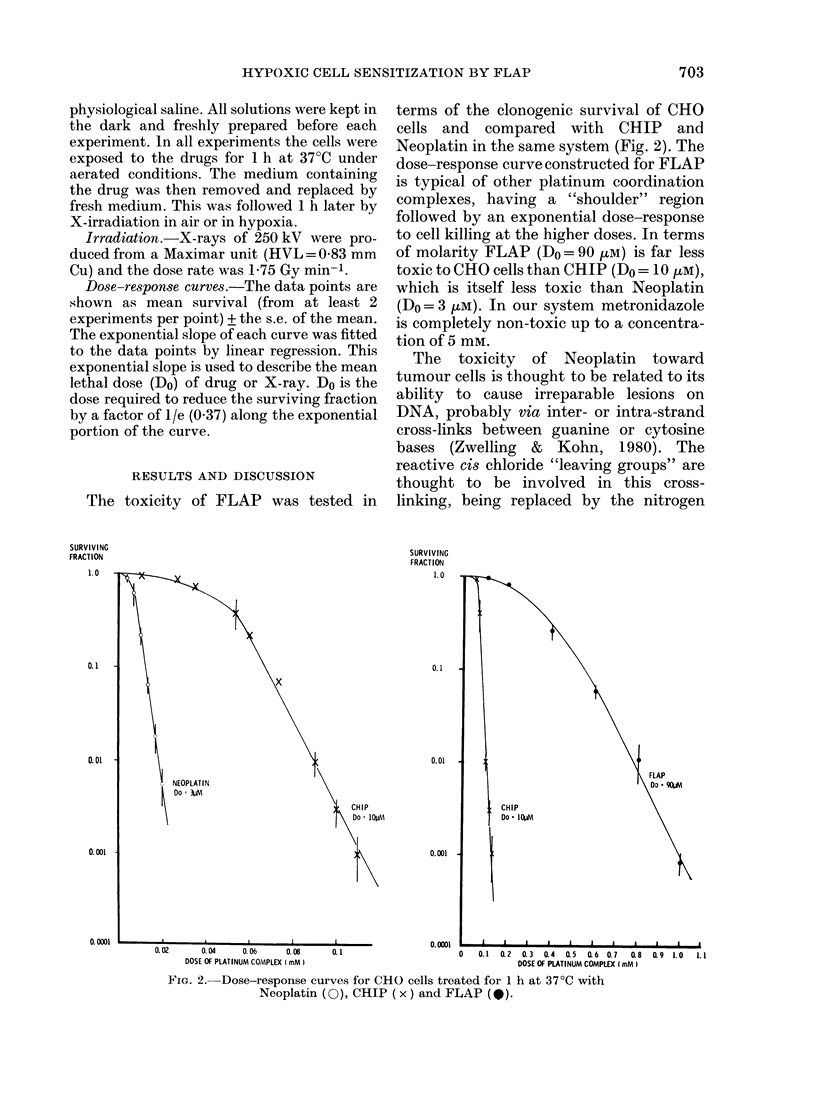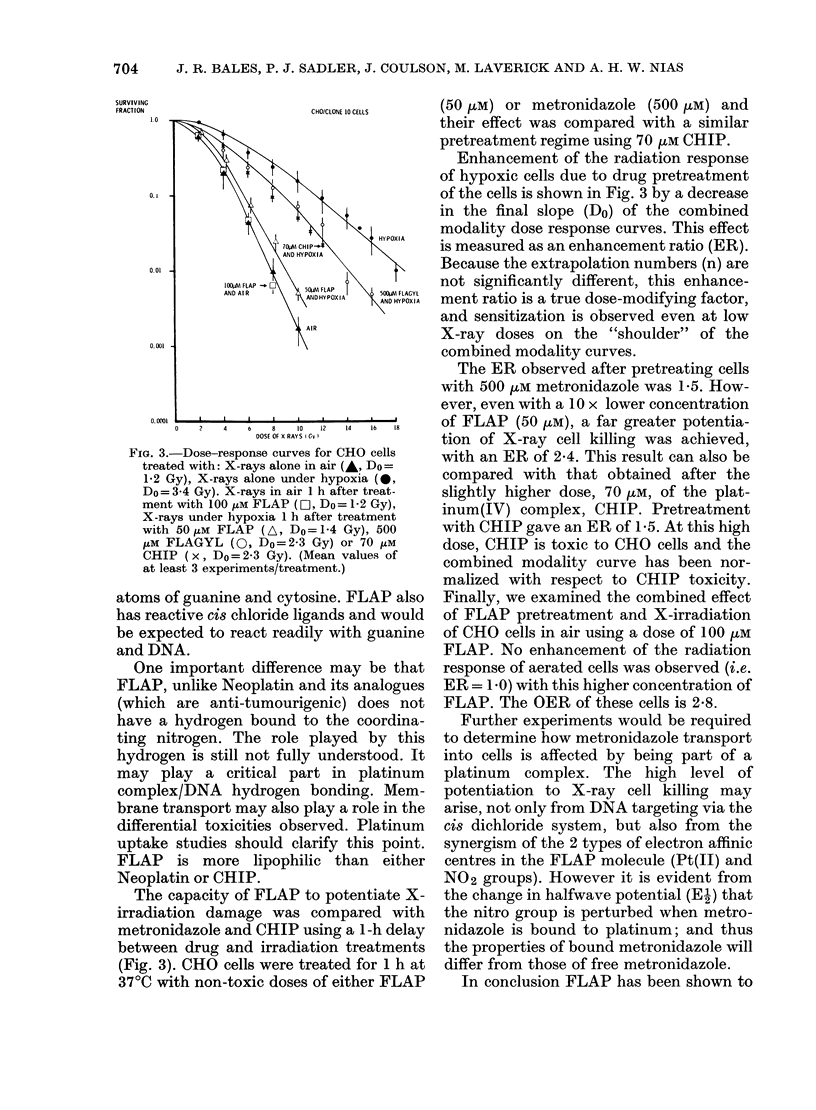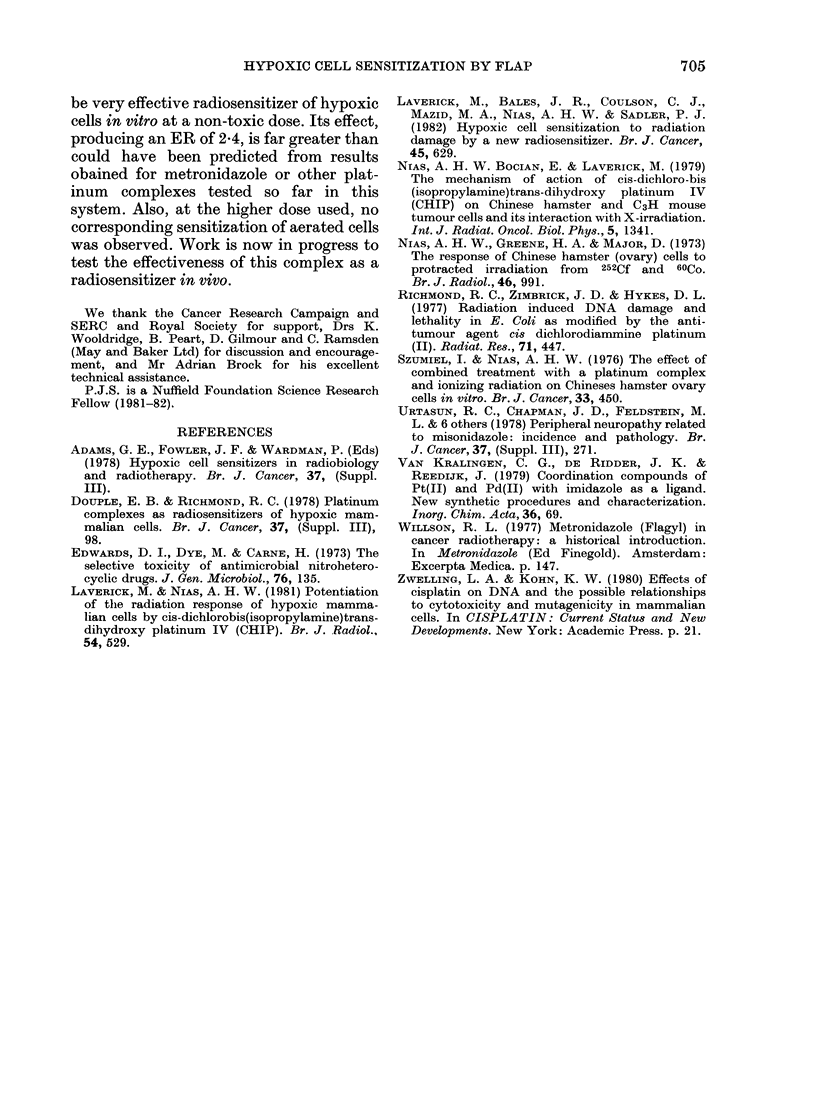# Hypoxic cell sensitization to radiation damage by a new radiosensitizer: cis-dichloro-bis(1-(2-hydroxyethyl)-2-methyl-5-nitroimidazole-N3)platinum(II) (flap).

**DOI:** 10.1038/bjc.1982.261

**Published:** 1982-11

**Authors:** J. R. Bales, P. J. Sadler, C. J. Coulson, M. Laverick, A. H. Nias

## Abstract

A new, stable platinum coordination complex (FLAP) containing the 5-nitroimidazole, metronidazole, has been prepared and characterized. The square-planar platinum(II) complex has two metronidazole molecules and two chlorine atoms in the cis configuration. The properties of FLAP differ significantly from metronidazole alone or other platinum complexes tested in the same system. It has a low toxicity towards Chinese hamster ovary cells and is a very effective radiosensitizer toward hypoxic cells in vitro: a one-h pretreatment with a non-toxic dose of 50 microM gave an enhancement ratio of 2.4. No potentiation of aerated cells to X-irradiation damage was observed after a similar schedule of pretreatment at the higher dose of 100 microM FLAP.


*Br. J. Cancer* (1982) **46**, 701

### HYPOXIC CELL SENSITIZATION TO RADIATION DAMAGE BY A NEW RADIOSENSITIZER: cis-DICHLORO-BIS(1-(2-HYDROXYETHYL)-2-METHYL-5-NITROIMIDAZOLE-$N^3$)PLATINUM(II) (FLAP)

J. R. BALES[a], P. J. SADLER[a], C. J. COULSON[b], M. LAVERICK[c] AND A. H. W. NIAS[c]

*From the [a]Department of Chemistry, Birkbeck College, Malet Street, London WC1E 7HX; the [b]Research Laboratories, May and Baker Ltd, Dagenham, Essex RM10 7XS and [c]the Richard Dimbleby Department of Cancer Research, St Thomas's Hospital Medical School, London SE1*

Received 11 March 1982 Accepted 21 July 1982

**Summary.—A new, stable platinum coordination complex (FLAP) containing the 5-nitroimidazole, metronidazole, has been prepared and characterized. The square-planar platinum(II) complex has two metronidazole molecules and two chlorine atoms in the *cis* configuration. The properties of FLAP differ significantly from metronidazole alone or other platinum complexes tested in the same system. It has a low toxicity towards Chinese hamster ovary cells and is a very effective radiosensitizer toward hypoxic cells *in vitro*: a one-h pretreatment with a non-toxic dose of 50 $\mu$M gave an enhancement ratio of 2·4. No potentiation of aerated cells to X-irradiation damage was observed after a similar schedule of pretreatment at the higher dose of 100 $\mu$M FLAP.**

THE ACHIEVEMENT of local control in the radiotherapy of tumours is often limited by regions of radioresistant but viable hypoxic cells. Hence there has been great interest in chemotherapeutic agents which can sensitize hypoxic tumour cells to ionizing radiation. Our aim was to design a new radiosensitizer, incorporating features of existing agents in a single compound. A combination of platinum and metronidazole, a nitroimidazole was chosen.

*Cis*-dichloro-diammine platinum(II) (Neoplatin) (Fig. 1a) is a potent anti-tumour agent now used in the clinic, but which also acts as a radiosensitizer both in mammalian and bacterial systems (Richmond *et al.*, 1977; Douple & Richmond, 1978). Another platinum(II) complex, *cis*-dichloro - bis(cyclopentylamine)platinum-(II) potentiates damage due to X-rays in aerated Chinese hamster ovary (CHO) cells (Szumiel & Nias, 1976) and the platinum(IV) complex *cis*-dichloro-bis(isopropylamine)*trans*-dihydroxy platinum(IV) (CHIP), exhibits potentiation of X-ray damage under hypoxic conditions (Laverick & Nias, 1981). Nitroheterocycles have also been widely studied as hypoxic cell radiosensitizers on account of the high electron affinity of the nitro group (Adams *et al.*, 1978; Willson, 1977). Thus, metronidazole or *Flagyl* (1-(2-hydroxyethyl)-2-methyl-5-nitroimidazole) (Fig. 1b) has undergone clinical trials (Urtasun *et al.*, 1978). Metronidazole is also preferentially cytotoxic to hypoxic cells and hence is widely used in the treatment of anaerobic bacterial infections (Edwards *et al.*, 1973).

Since the nitrogen at the 3-position of metronidazole bears a lone pair of electrons, it was decided to attempt the synthesis of a complex of the type, Pt(II)-(metronidazole)$_2$Cl$_2$ (Fig. 1c), preferably with the chloride ligands in the *cis* configuration. It was hoped that, as the complex was neutral, it would readily pass through cell membranes. As in the case of Neoplatin, it has 2 potentially reactive *cis*

Correspondence to Professor A. H. W. Nias.

FIG. 1.—(a) *Cis*-dichloro-diammine platinum(II); (b) 1-(2-hydroxyethyl)-2-methyl-5-nitroimidazole; (c) *cis*-dichloro-bis(1-(2-hydroxyethyl)-2-methyl-5-nitroimidazole-$N^3$)platinum(II).

chloride ligands which might enable it specifically to attack guanine-cytosine rich regions of DNA and bring both electron affinic centres (platinum and $NO_2$) near to DNA, the target of radiation damage. However, it was predicted that, unlike Neoplatin, the new complex would *not* be an anti-tumour agent in its own right, since the nitrogens coordinated to platinum do not bear hydrogen atoms. Indeed, *cis* - dichloro - bis(imidazolo)platinum(II) and substituted imidazole complexes (without $NO_2$ groups) have been reported to exhibit only marginal cytostatic activity (Van Kralingen *et al.*, 1979).

We describe the successful synthesis and characterization of *cis*-platinum(metronidazole)$_2$Cl$_2$ (FLAP) and its radiosensitizing property when given as a pretreatment to hypoxic cells in culture. The enhancement ratio observed (2·4) was far greater than could have been predicted from results obtained from either metronidazole alone or other platinum complexes tested previously in the same system. A brief report of this work has already appeared (Laverick *et al.*, 1982).

### MATERIALS AND METHODS

*Preparation and characterization of FLAP.*—Full chemical details will be published elsewhere; a summary is given here. *Cis*-platinum(metronidazole)$_2$Cl$_2$ was prepared, in high yield, by reacting $K_2PtCl_4$ with 2 molar-equivalents of metronidazole in water. It can be recrystallized from acetone-water, although it has a high level of purity after the initial preparation. The purity was confirmed by elemental analysis (C, H, N, Cl, and Pt) and proton nuclear magnetic resonance spectroscopy. Spin–spin coupling between the metronidazole $C_4$-H and $C_2$-methyl hydrogen atoms and $^{195}Pt$, confirmed that platinum was coordinated at the $N_3$ nitrogen. The complex is a stable yellow solid (m.p. 180–182°C), sparingly soluble in water (*ca.* 80 $\mu$M) but more soluble in acetone, dimethylformamide, methoxyethanol and various glycols. The polarographic halfwave potential ($E\frac{1}{2}$) recorded in phosphate buffer at pH 7 *vs* an Ag/AgCl reference, was $-0{\cdot}27$ V. This can be compared with a value of $-0{\cdot}47$ V for metronidazole alone under similar conditions.

The *cis* configuration of the complex was confirmed by X-ray crystallography (Bales *et al.* unpublished). The 2 chlorine and 2 nitrogen atoms form a square-plane around platinum, the planes of the metronidazole rings being approximately perpendicular to this plane, tilted slightly with respect to each other.

*Cell culture.*—CHO cells were grown in monolayer culture in HEPES—buffered Minimal Essential Medium (MEM) supplemented with 15% calf serum, non-essential amino acids and L-glutamine. Other methods of cell culture together with the determination of cell survival by their colony forming ability after 5 days growth at 37°C have been described previously (Szumiel & Nias, 1976; Nias *et al.*, 1979). Hypoxia was produced in cells by the method of Nias *et al.* (1973).

*Drug treatment.*—FLAP was dissolved at a concentration of 10 mg ml$^{-1}$ in propylene glycol with warming and diluted with MEM. Metronidazole was dissolved in physiological saline at a concentration of 25 mM. Stock solutions of CHIP and Neoplatin (supplied by Johnson Matthey Ltd) were prepared at 1 mg ml$^{-1}$ and 200 $\mu$g ml$^{-1}$ respectively, in physiological saline. All solutions were kept in the dark and freshly prepared before each experiment. In all experiments the cells were exposed to the drugs for 1 h at 37°C under aerated conditions. The medium containing the drug was then removed and replaced by fresh medium. This was followed 1 h later by X-irradiation in air or in hypoxia.

*Irradiation*.—X-rays of 250 kV were produced from a Maximar unit (HVL = 0·83 mm Cu) and the dose rate was 1·75 Gy $min^{-1}$.

*Dose–response curves*.—The data points are shown as mean survival (from at least 2 experiments per point) ± the s.e. of the mean. The exponential slope of each curve was fitted to the data points by linear regression. This exponential slope is used to describe the mean lethal dose ($D_0$) of drug or X-ray. $D_0$ is the dose required to reduce the surviving fraction by a factor of 1/e (0·37) along the exponential portion of the curve.

### RESULTS AND DISCUSSION

The toxicity of FLAP was tested in terms of the clonogenic survival of CHO cells and compared with CHIP and Neoplatin in the same system (Fig. 2). The dose–response curve constructed for FLAP is typical of other platinum coordination complexes, having a "shoulder" region followed by an exponential dose–response to cell killing at the higher doses. In terms of molarity FLAP ($D_0 = 90$ μM) is far less toxic to CHO cells than CHIP ($D_0 = 10$ μM), which is itself less toxic than Neoplatin ($D_0 = 3$ μM). In our system metronidazole is completely non-toxic up to a concentration of 5 mM.

The toxicity of Neoplatin toward tumour cells is thought to be related to its ability to cause irreparable lesions on DNA, probably *via* inter- or intra-strand cross-links between guanine or cytosine bases (Zwelling & Kohn, 1980). The reactive *cis* chloride "leaving groups" are thought to be involved in this cross-linking, being replaced by the nitrogen

FIG. 2.—Dose–response curves for CHO cells treated for 1 h at 37°C with Neoplatin (○), CHIP (×) and FLAP (●).

Fig. 3.—Dose–response curves for CHO cells treated with: X-rays alone in air (▲, $D_0$ = 1·2 Gy), X-rays alone under hypoxia (●, $D_0$ = 3·4 Gy). X-rays in air 1 h after treatment with 100 $\mu$M FLAP (□, $D_0$ = 1·2 Gy), X-rays under hypoxia 1 h after treatment with 50 $\mu$M FLAP (△, $D_0$ = 1·4 Gy), 500 $\mu$M FLAGYL (○, $D_0$ = 2·3 Gy) or 70 $\mu$M CHIP (×, $D_0$ = 2·3 Gy). (Mean values of at least 3 experiments/treatment.)

atoms of guanine and cytosine. FLAP also has reactive *cis* chloride ligands and would be expected to react readily with guanine and DNA.

One important difference may be that FLAP, unlike Neoplatin and its analogues (which are anti-tumourigenic) does not have a hydrogen bound to the coordinating nitrogen. The role played by this hydrogen is still not fully understood. It may play a critical part in platinum complex/DNA hydrogen bonding. Membrane transport may also play a role in the differential toxicities observed. Platinum uptake studies should clarify this point. FLAP is more lipophilic than either Neoplatin or CHIP.

The capacity of FLAP to potentiate X-irradiation damage was compared with metronidazole and CHIP using a 1-h delay between drug and irradiation treatments (Fig. 3). CHO cells were treated for 1 h at 37°C with non-toxic doses of either FLAP (50 $\mu$M) or metronidazole (500 $\mu$M) and their effect was compared with a similar pretreatment regime using 70 $\mu$M CHIP.

Enhancement of the radiation response of hypoxic cells due to drug pretreatment of the cells is shown in Fig. 3 by a decrease in the final slope ($D_0$) of the combined modality dose response curves. This effect is measured as an enhancement ratio (ER). Because the extrapolation numbers (n) are not significantly different, this enhancement ratio is a true dose-modifying factor, and sensitization is observed even at low X-ray doses on the "shoulder" of the combined modality curves.

The ER observed after pretreating cells with 500 $\mu$M metronidazole was 1·5. However, even with a 10 × lower concentration of FLAP (50 $\mu$M), a far greater potentiation of X-ray cell killing was achieved, with an ER of 2·4. This result can also be compared with that obtained after the slightly higher dose, 70 $\mu$M, of the platinum(IV) complex, CHIP. Pretreatment with CHIP gave an ER of 1·5. At this high dose, CHIP is toxic to CHO cells and the combined modality curve has been normalized with respect to CHIP toxicity. Finally, we examined the combined effect of FLAP pretreatment and X-irradiation of CHO cells in air using a dose of 100 $\mu$M FLAP. No enhancement of the radiation response of aerated cells was observed (*i.e.* ER = 1·0) with this higher concentration of FLAP. The OER of these cells is 2·8.

Further experiments would be required to determine how metronidazole transport into cells is affected by being part of a platinum complex. The high level of potentiation to X-ray cell killing may arise, not only from DNA targeting via the *cis* dichloride system, but also from the synergism of the 2 types of electron affinic centres in the FLAP molecule (Pt(II) and $NO_2$ groups). However it is evident from the change in halfwave potential ($E\frac{1}{2}$) that the nitro group is perturbed when metronidazole is bound to platinum; and thus the properties of bound metronidazole will differ from those of free metronidazole.

In conclusion FLAP has been shown to be very effective radiosensitizer of hypoxic cells *in vitro* at a non-toxic dose. Its effect, producing an ER of 2·4, is far greater than could have been predicted from results obained for metronidazole or other platinum complexes tested so far in this system. Also, at the higher dose used, no corresponding sensitization of aerated cells was observed. Work is now in progress to test the effectiveness of this complex as a radiosensitizer *in vivo*.

We thank the Cancer Research Campaign and SERC and Royal Society for support, Drs K. Wooldridge, B. Peart, D. Gilmour and C. Ramsden (May and Baker Ltd) for discussion and encouragement, and Mr Adrian Brock for his excellent technical assistance.

P.J.S. is a Nuffield Foundation Science Research Fellow (1981–82).

### REFERENCES

Adams, G. E., Fowler, J. F. & Wardman, P. (Eds) (1978) Hypoxic cell sensitizers in radiobiology and radiotherapy. *Br. J. Cancer*, **37**, (Suppl. III).

Douple, E. B. & Richmond, R. C. (1978) Platinum complexes as radiosensitizers of hypoxic mammalian cells. *Br. J. Cancer*, **37**, (Suppl. III), 98.

Edwards, D. I., Dye, M. & Carne, H. (1973) The selective toxicity of antimicrobial nitroheterocyclic drugs. *J. Gen. Microbiol.*, **76**, 135.

Laverick, M. & Nias, A. H. W. (1981) Potentiation of the radiation response of hypoxic mammalian cells by cis-dichlorobis(isopropylamine)trans-dihydroxy platinum IV (CHIP). *Br. J. Radiol.*, **54**, 529.

Laverick, M., Bales, J. R., Coulson, C. J., Mazid, M. A., Nias, A. H. W. & Sadler, P. J. (1982) Hypoxic cell sensitization to radiation damage by a new radiosensitizer. *Br. J. Cancer*, **45**, 629.

Nias, A. H. W. Bocian, E. & Laverick, M. (1979) The mechanism of action of cis-dichloro-bis (isopropylamine)trans-dihydroxy platinum IV (CHIP) on Chinese hamster and C3H mouse tumour cells and its interaction with X-irradiation. *Int. J. Radiat. Oncol. Biol. Phys.*, **5**, 1341.

Nias, A. H. W., Greene, H. A. & Major, D. (1973) The response of Chinese hamster (ovary) cells to protracted irradiation from $^{252}Cf$ and $^{60}Co$. *Br. J. Radiol.*, **46**, 991.

Richmond, R. C., Zimbrick, J. D. & Hykes, D. L. (1977) Radiation induced DNA damage and lethality in *E. Coli* as modified by the antitumour agent *cis* dichlorodiammine platinum (II). *Radiat. Res.*, **71**, 447.

Szumiel, I. & Nias, A. H. W. (1976) The effect of combined treatment with a platinum complex and ionizing radiation on Chineses hamster ovary cells *in vitro*. *Br. J. Cancer*, **33**, 450.

Urtasun, R. C., Chapman, J. D., Feldstein, M. L. & 6 others (1978) Peripheral neuropathy related to misonidazole: incidence and pathology. *Br. J. Cancer*, **37**, (Suppl. III), 271.

Van Kralingen, C. G., de Ridder, J. K. & Reedijk, J. (1979) Coordination compounds of Pt(II) and Pd(II) with imidazole as a ligand. New synthetic procedures and characterization. *Inorg. Chim. Acta*, **36**, 69.

Willson, R. L. (1977) Metronidazole (Flagyl) in cancer radiotherapy: a historical introduction. In *Metronidazole* (Ed Finegold). Amsterdam: Excerpta Medica. p. 147.

Zwelling, L. A. & Kohn, K. W. (1980) Effects of cisplatin on DNA and the possible relationships to cytotoxicity and mutagenicity in mammalian cells. In *CISPLATIN: Current Status and New Developments*. New York: Academic Press. p. 21.